# Overcoming diffusion-related limitations in semiconductor defect imaging with phonon-plasmon-coupled mode Raman scattering

**DOI:** 10.1038/s41377-018-0016-y

**Published:** 2018-06-20

**Authors:** Changkui Hu, Qiong Chen, Fengxiang Chen, T. H. Gfroerer, M. W. Wanlass, Yong Zhang

**Affiliations:** 1 University of North Carolina at Charlotte, Charlotte, NC 28223 USA; 2 Wuhan University of Technology, Wuhan, Hubei 430070 China; 3 Davidson College, Davidson, NC 28035 USA; 4 National Renewable Energy Laboratory, Golden, CO 80401 USA

## Abstract

Carrier diffusion is of paramount importance in many semiconductor devices, such as solar cells, photodetectors, and power electronics. Structural defects prevent such devices from reaching their full performance potential. Although a large carrier diffusion length indicates high material quality, it also implies increased carrier depletion by an individual extended defect (for instance, a dislocation) and obscures the spatial resolution of neighboring defects using optical techniques. For commonly utilized photoluminescence (PL) imaging, the spatial resolution is dictated by the diffusion length rather than by the laser spot size, no matter the spot is at or below the diffraction limit. Here, we show how Raman imaging of the LO phonon-plasmon-coupled mode can be used to recover the intrinsic spatial resolution of the optical system, and we demonstrate the effectiveness of the technique by imaging defects in GaAs with diffraction-limited optics, achieving a 10-fold improvement in resolution. Furthermore, by combining Raman and PL imaging, we can independently and simultaneously determine the spatial dependence of the electron density, hole density, radiative recombination rate, and non-radiative recombination rate near a dislocation-like defect, which has not been possible using other techniques.

## Imaging: Characterising defects paves way for better performing electronic devices

A new technique for characterising defects in semiconductor materials could lead to better performing electronic devices. Understanding various defects and their effects on a semiconductor’s properties and behavior is key to improving the performance of electronic devices. The spatial resolution of commonly used imaging techniques like photoluminescence imaging, however, are limited by carrier diffusion length, which restricts their ability to characterize different defects. By combining Raman and photoluminescence imaging, Yong Zhang and colleagues from the University of North Carolina at Charlotte in the United States, in collaboration with fellow Chinese and American researchers, were able to observe the impact of a defect on the properties of gallium arsenide, allowing them to more effectively characterize the defect and its impact previously impractical. The work could lead to better performing solar cells, photodetectors, and power electronics devices.

## Introduction

While both point defects (PDs) and extended defects (EDs) may yield qualitatively similar effects^1–3^, for example, depletion of carriers that are supposed to generate radiative recombination or carry electrical current, they often play competitive roles in affecting the device performance^4,5^. For instance, PDs suppress carrier diffusion and thus may diminish the impact of EDs. It is relatively easy to saturate PDs in a moderately high-quality material with a high carrier density, but an ED tends to introduce a very high density of defect states that are practically impossible to saturate by simply increasing the carrier injection level. In fact, before being saturated by increasing illumination power, a dislocation can mutate into a defect network that is more detrimental than the original form^5^. Furthermore, different EDs may behave very differently, with most EDs being detrimental to various degrees while some are benign to photogenerated carriers^6^. Therefore, it is important to distinguish and investigate EDs individually and, ultimately, to identify their atomistic structures.

To quantitatively investigate the impact of a defect, one would need to first locate it on a macroscopic device. Photoluminescence (PL) imaging is the natural technique to use because of the relative ease of the experiment. Various PL-based imaging techniques with either diffraction-limit or below-diffraction-limit spatial resolution have been developed for probing nanostructures and individual molecules^7–10^, where carrier diffusion is tolerable or irrelevant. In a bulk semiconductor, carrier diffusion is an important aspect of carrier transport, particularly when performing operando studies of defects in a device with either photogenerated or electrically injected carriers. For a radiative defect, one can perform PL/EL imaging using the spectral properties of the defect (if it can be spectroscopically resolved from the band edge emission). In such cases, the spatial resolution is determined by the optical system and is not affected by carrier diffusion. Examples of radiative defects that facilitate this approach include nitrogen vacancy centers in diamond^11^ and nitrogen pairs in GaAs^12^. However, for non-radiative defects such as dislocations in GaP^4^ and dislocations and grain boundaries in CdTe^6,13,14^, the common approach is to image the band edge PL/EL to reveal the location where the luminescence signal is weakened by defect-induced carrier depletion. In this situation, the spatial resolution is drastically degraded relative to the capability of the optical system when the carrier diffusion length (DL) is greater than the optically defined spatial resolution. In the PL image, the defect may visually appear to be much larger that its actual size because of carrier depletion in the surrounding regions over a distance comparable to the DL from the defect site (a non-local effect)^5,15,16^. It was shown recently that when an ED presents a space-charge field, second harmonic generation is enhanced at the defect site, which yields a significantly more localized intensity distribution than PL^17^. However, not all defects can offer such enhancement^17^. Since the most basic and detrimental characteristic of an ED is the depletion of carriers, a technique that is highly sensitive to the carrier density is desirable.

Since atomic vibrations are expected to be altered in the vicinity of a defect^18^, Raman scattering can in principle be used to probe defects. Moreover, Raman spectroscopy is not susceptible to carrier diffusion when excited in the transparent region. Indeed, Raman imaging has long been used to characterize mesoscopic or macroscopic structural inhomogeneity in semiconductors, such as GaAs^19–21^, where carrier diffusion is either irrelevant or negligible. However, because Raman efficiencies are typically many orders of magnitude lower than the PL efficiency^22^ and defect-induced perturbations of vibrational properties tend to be very local, it is impractical in most realistic situations to probe individual microscopic defects with conventional Raman spectroscopy, even when employing a below-diffraction-limit technique. Since the size of a defect core for a simple dislocation is on the order of a few nanometers^23^ and the conventional diffraction-limit beam size is several hundreds of nanometers, a reduction in beam size of at least two orders of magnitude is required to match the defect core size to directly locate the defect using its vibrational signature. However, to obtain the same signal level, one requires an increase in excitation density of four orders of magnitude, which is sufficient (e.g., >10^6^ W/cm^2^) to induce a structural change in the dislocation defect, even for a material with fairly strong chemical bonding such as GaAs^5^. Therefore, for a high-quality sample, that is, with a long DL, a new technique is required to better resolve non-radiative defects in the presence of diffusing carriers. The LO phonon-plasmon (LOPP) Raman scattering technique demonstrated in this work enables us to use a diffraction-limited beam (sub-µm) to achieve a µm-scale point spread function in a sample with a DL ≈ 20 µm. We also show that further improvement is possible by using sub-diffraction-limit optics^7^.

Despite the diffraction limit, it is possible to use fluorescence imaging to distinguish and resolve individual molecules within a single diffraction-limit volume if they have different spectral or temporal properties^8–10^. Akin to these approaches, to suppress the impact of diffusion on non-radiative defect imaging, it is desirable to utilize a beyond-diffusion-limit (BDL) technique using a spectroscopic signature that is distinguishable between the defect and defect-free sites. Since the carrier density varies rapidly in the vicinity of an ED, a spectroscopic feature with superlinear density dependence can enable one to transcend diffusive limitations. LOPP Raman scattering offers the desired characteristic of strong nonlinear density dependence, in sharp contrast to the weaker carrier dependence of PL. Note that this technique also requires carrier diffusion to generate a non-uniform carrier density distribution, which makes it possible to locate a defect whose physical size is approximately one-hundredth of the size of the optical beam.

The coupling of the LO phonon with free electrons (plasmons) arising from doping leads to the formation of the hybrid modes L_+_ and L_−_, and the frequency and intensity of the Raman signal of L_+_ are very sensitive to the carrier density^24^. The same effect is observed for photogenerated electron–hole plasmas^25–27^. The underlying physics of the drastically improved spatial resolution can be explained as follows: (1) the Raman frequency *ν*_+_(*n*) = *v*_LO_ + *α*_1_*n* + *α*_2_*n*^2^ … is a superlinear function (i.e., *α*_1_ > 0 and *α*_2_ > 0), where *n* is the carrier density; (2) the Raman cross-section *R*(*n*) decreases with increasing *n*; and most importantly, (3) a Gaussian or Lorentzian-type lineshape function *f*[(δ*ν*/*w*)^2^] affords a very strong dependence of the Raman intensity on the frequency shift, where δ*ν* = *ν*_+_(*n*) − *ν*_+_(*n*_0_), with *ν*_+_(*n*_0_) = *ν*_0_ being the L_+_ mode frequency at the defect site with carrier density *n*_0_, and *w* is the full-width at half-maximum, or FWHM. Under excitation, the carriers are mostly depleted by rapid non-radiative recombination at the defect site (i.e., *n*_0_ << *n*), and thus the Raman frequency *ν*_0_ is expected to remain close to *ν*_LO_. However, as soon as we move away from the defect, a moderate excitation density will be adequate to induce a peak shift δ*ν* comparable to the intrinsic LO mode linewidth (~2 cm^−1^ at room temperature^28^). The combination of these properties results in the Raman signal at *ν*_0_ ≈ *ν*_LO_ exhibiting a much stronger dependence on *n*, and thus a much more rapid spatial variation than that of PL.

Though the spatial dependence of the PL intensity near an ED can be measured by PL imaging^5,14,16^, it is not possible to independently analyze the spatial dependence of the carrier density *n*(*r*), radiative recombination rate *W*_r_(*r*), and non-radiative recombination *W*_nr_(*r*) because the measured *I*_PL_(*r*) = *n*(*r*)*W*_r_(*r*) is a product of two quantities, and *W*_nr_(*r*) is implicitly involved. Even if spatial and time-resolved imaging are performed simultaneously, one still cannot separate *W*_r_(*r*) and *W*_nr_(*r*) because the local PL decay time *τ*(*r*) is given by *τ*(*r*)^−1^ = *W*_r_(*r*) + *W*_nr_(*r*). Since *W*_r_(*r*) is not experimentally accessible, it is typically assumed to be constant throughout the material^14^. However, since LOPP Raman imaging provides a straightforward method to obtain the spatial variation *n*(*r*) near the defect, combined with PL mapping, we are able to obtain spatial profiles for both the radiative and non-radiative recombination rates *W*_r_(*r*) and *W*_nr_(*r*) near a defect. This opens up a route towards the development of new diagnostic techniques for semiconductor materials and devices.

## Results

Multiple GaInP/GaAs/GaInP double heterostructures were used to examine the general applicability of the approach under different conditions. The results of three samples, S1, S2, and S3, are reported. These samples have very low dislocation-type defect densities (approximately a few hundred per cm^−2^)^5,16^. All experiments were conducted at room temperature using a confocal Raman microscope with a diffraction-limited excitation spot size of approximately 720 nm in diameter. Further details about the samples and measurements can be found in the Materials and methods section.

**Fig. 1:**
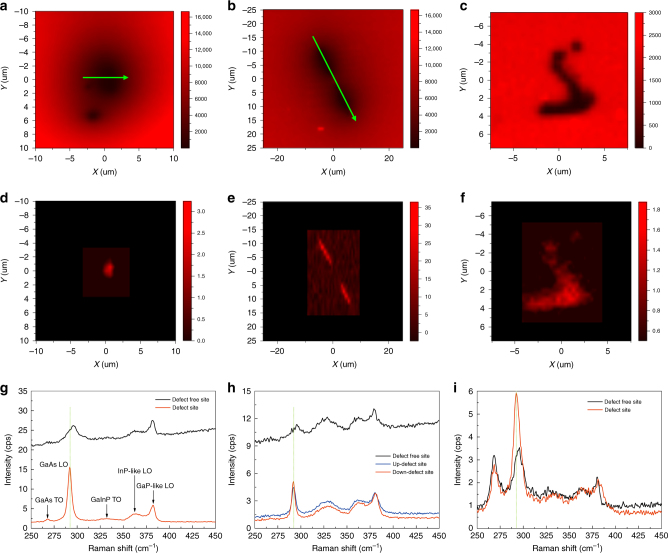
PL and Raman images near a defect and typical Raman spectra measured at and away from the defect for three GaAs samples. **a**–**c** PL images, **d**–**f** Raman images, **g**–**i** Raman spectra (data were not obtained in the black outer region). Left column—S1 (at 3.7 × 10^4^ W/cm^2^); central and right column—S2 and S3 (at 5.6 × 10^4^ W/cm^2^)

Fig. 1 compares the PL and Raman imaging results near an isolated defect in each of the three samples. The PL images use the signal at 870 nm (20 nm bandwidth), and the Raman images use the LO mode (0.5 cm^−1^ bandwidth) of the defect site. For S1, the PL image near the defect, Fig. 1a, shows a dark area that is much larger than the laser spot size because of diffusion. The DL derived from the PL image is ~20 µm (following the method of Chen et al.^16^). Note that the effective defect impact range already appears to be significantly smaller than that given by the DL because of the improved spatial resolution of the raster scan mode (local excitation and local collection, L/L) compared to the uniform illumination mode (uniform illumination and local detection, U/L)^16^. However, the effective impact range of the defect in the Raman image in Fig. 1d is further reduced to just over 1 µm due to the LO phonon-plasmon (LOPP) coupling effect. Because of the above-bandgap excitation, the presence of a steady-state carrier density leads to the formation of a phonon-plasmon complex^25^, but the effect diminishes approaching the defect. Fig. 1g compares Raman spectra at the defect and defect-free sites, where the GaAs LO or L_+_ mode is significantly blueshifted, broadened, and weakened at the defect-free site (the background difference is due to the tail of the PL signal) compared to the defect site, which explains the superior spatial resolution in Raman imaging. Note that there is little change in the GaInP-related modes^29^, confirming that the effect is indeed originated in the GaAs layer. The stronger and sharper Raman mode at the defect site might seem counterintuitive and opposite to what one would expect for a defect: showing a weaker and broadened Raman peak^18^. However, until the beam size is substantially reduced, Raman imaging does not probe the phonon mode of the microscopic defect itself. Rather, the Raman signal is generated from the excitation volume of the laser beam, and reflect the impact of the defect on the surrounding bulk-like material. Thus, the findings are exactly as expected for the LOPP mode^30^. The results of S1 indicate that Raman imaging overcomes the diffusion-related limitations of PL imaging and uncovers the diffraction-limited point spread function of the microscopic defect. Note that the ability to suppress diffusion-induced blurring is not simply because Raman is insensitive to diffusion but because the joint effect of LOPP coupling and diffusion causes the LO phonon Raman scattering to exhibit a superlinear dependence on the carrier density.

For S2, a pair of neighboring defects is examined to verify the improvement in spatial resolution. In PL imaging, with a DL ≈24 µm, the two defects were barely resolvable under U/L mode^16^, but much better resolved under L/L mode, even though the diffusion effect remains significant, as shown in Fig. 1b. However, with Raman imaging, the spatial resolution is virtually unaffected by the diffusion, as shown in Fig. 1e. The Raman spectra in Fig. 1h exhibit similar contrast between the defect and defect-free site, as observed in S1. For S3, since the lateral diffusion is weaker, the contrast for the defect impact range between the PL image in Fig. 1c and the Raman image in Fig. 1f is not as substantial, but significant differences between the defect and defect-free site remain in their Raman spectra, as seen in Fig. 1i. Clearly, Raman mapping is a generally applicable and effective tool for realizing BDL defect imaging in semiconductors under different sample conditions.

**Fig. 2:**
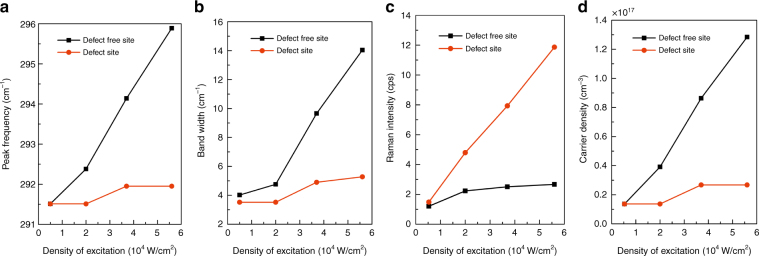
Comparison of the L_+_ mode characteristic vs. excitation density for the defect and defect-free sites for S1. **a** peak frequency, **b** bandwidth, **c** intensity, and **d** carrier density

Fig. 2a–c plot the excitation power (*P*) density dependence, (0.53 – 5.6) × 10^4^ W/cm^2^, for the peak frequency (*ν*_m_), linewidth (*w*), and peak intensity (*I*_m_) of the L_+_ mode of the defect and defect-free sites in S1. The results are qualitatively similar for the other samples. At the defect site, the variations are minimal for *ν*_m_ and *w*, but *I*_m_ steadily increases with increasing *P*, which indicates that the carrier density at the defect site remains low because it is not possible to saturate such a dislocation-type defect before altering the defect structure should an even higher power be used^5^. In contrast, at the defect-free site, *ν*_m_ and *w* steadily increase with increasing *P*, while *I*_m_ increases only slightly, as expected for the L_+_ mode. Fig. 2d shows the electron density *n* vs. *P*, using the standard formula *ν*_+_(*n*)^24^ with the following parameters: *hν*_LO_ = 291.5 cm^−1^, *hν*_TO_ = 268.0 cm^−1^, *ϵ*_0_ =12.8, and *ϵ*_∞_=10.86. Sublinear dependence, (1.37 − 2.68) × 10^16^ cm^−3^, is found at the defect site due to depletion; nearly linear dependence, 1.37 × 10^16^ to 1. 28 × 10^17^ cm^−3^, is observed at the defect-free site.

## Discussion

**Fig. 3:**
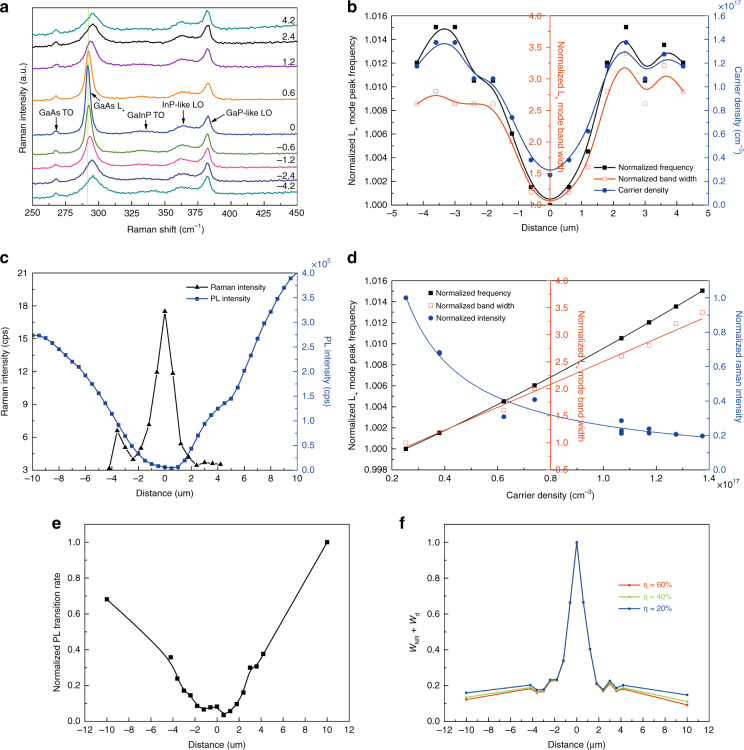
Raman and PL properties near the defect in S1 shown in Fig. 1a, examined along a line passing along the center of the defect (at 5.6 × 10^4^ W/cm^2^). **a** Raman spectra at different displacements from the defect (in μm, increasing value from left to right). **b** Spatial profiles of the L_+_ mode frequency and width (both normalized to the defect site), and carrier density (right axis). **c** Spatial profiles of the Raman intensity at *ν*_0_ and PL intensity at the bandgap energy. **d** L_+_ mode peak frequency, width, and intensity at *ν*_0_ (normalized to the defect site) vs. carrier density. **e** Normalized radiative recombination rate. **f** Normalized non-radiative recombination loss rate

Fig. 3 examines the spatial dependence obtained using a line scan across the center of the defect, as marked in Fig. 1a, for the defect in S1. Fig. 3a shows the evolution of the Raman spectrum, revealing drastic changes in the L_+_ mode near the defect. Fig. 3b shows the spatial dependence of the L_+_ mode peak frequency and linewidth, as well as the carrier density. All return to their background values within approximately 3 μm. Fig. 3c contrasts the spatial variation in the Raman intensity at *ν*_0_ and the PL intensity at the bandgap energy. The PL intensity reaches the background value within approximately 10 μm, which is roughly equivalent to half of the DL, in the L/L mode, but the Raman intensity reaches the background level within approximately 2 μm, which is approximately 1/10 of the DL. Given that the probe beam size is approximately 0.7 μm, the rapid change in Raman intensity is the dominant mechanism that affords the high spatial resolution. Fig. 3d depicts the L_+_ mode frequency, linewidth, and Raman intensity at *ν*_0_ against the electron density. These three parameters ultimately determine the spatial resolution. The frequency exhibits a nearly linear dependence in this density range: *ν*_+_ = *ν*_LO_ + 3.13 × 10^−17^ *n* + 4.78 × 10^−35^ *n*^2^, as predicted by the formula for the coupled mode^24^. The (normalized) Raman intensity exhibits a dependence of *I*(*n*) = 1/(1+5.31*n*^1.47^), whereas the (normalized) linewidth shows a near linear dependence, *w* = 0.388 + 2.116 × 10^−17^ *n*. Using the obtained *I*(*n*) dependence and assuming an ideal carrier profile based on the Bessel *K* function^16^, we can estimate the theoretical limit for the spatial resolution when the measurement is not constrained by the diffraction limit. Assuming a DL of 20 μm and a defect with a core size of 10 nm, within which the carrier density is zero and beyond which the carrier density is described by the solution to the Bessel function^16^, the simulation of the spatial profile of the Raman intensity yields an FWHM of approximately 100 nm or 1/200 of the DL. Comparing this estimate to the ~2 µm width in Fig. 3c, we infer that a further improvement in the spatial resolution is practically feasible if a sub-diffraction-limit excitation source is used.

We next discuss how the radiative and non-radiative recombination rates near a defect are impacted. It is rarely possible to obtain such information. Fig. 3e plots the (normalized) PL transition rate *W*_r_(*r*) = *I*_PL_(*r*)/*n*(*r*), which turns out to be strongly spatially dependent, rather than constant as one might expect^14^. This result reveals very important information about the defect that is not readily available from PL mapping alone in either CW or time-resolved mode. The electron density distribution in Fig. 3b is highly localized, which implies the defect is a hole trap rather than an electron trap because electrons tend to have a much longer DL^5^. This assertion is corroborated by the previous finding that the defect impact area was drastically increased after the defect structure was altered by illumination at higher power^5^. For *W*_r_ = *γp*, with *p* being the hole concentration and *γ* the radiative recombination coefficient (a constant), *W*_r_(*r*) actually reflects the hole distribution including diffusion. As a hole trap, the defect only depletes the electrons over a short range of a couple of μm near the defect core. The mismatch in the electron and hole distributions implies the formation of a polarization electrical field near the defect. The imbalance between the two charge distributions suggests that the diffusion is non-ambipolar^31^. Therefore, the carrier diffusion process could be much more complicated than we have originally considered^16^. One can further infer information about the non-radiative recombination rate *W*_nr_(*r*) near the defect, as plotted in Fig. 3f for a normalized non-radiative recombination rate. Because the carrier diffusion away from the excitation site represents an additional loss mechanism under the L/L mode^16,32^, we can write the total loss as *W*_loss_ = *W*_nr_ + *W*_d_, with *W*_nr_ being the genuine non-radiative recombination loss associated with both uniformly distributed PDs and the particular dislocation and *W*_d_ being an effective rate for the diffusion loss. From the rate equation, we can show that *W*_loss_=*G*[1 − *η*_PL_*I*_PL_(*r*)/*I*_PL_(∞)]/*n*(*r*), where *G* is the generation rate, *I*_PL_(*r*) and *n*(*r*) are the PL intensity and electron density at a distance *r* from the defect, respectively, and *η*_PL_ is the PL efficiency far away from the defect (*η*_PL_ ≤ 1 due to non-radiative loss through PDs and diffusion). Fig. 3f shows that *W*_loss_ is very high in a small region near the defect, similar to the electron distribution in Fig. 3b, but quickly drops off beyond that to a background level that is only weakly dependent on the choice of *η*_PL_, where *W*_d_ likely dominates. For the first time, we can distinguish the radiative and non-radiative recombination processes near an individual defect by taking advantage of the combined power of PL and Raman mapping.

**Fig. 4:**
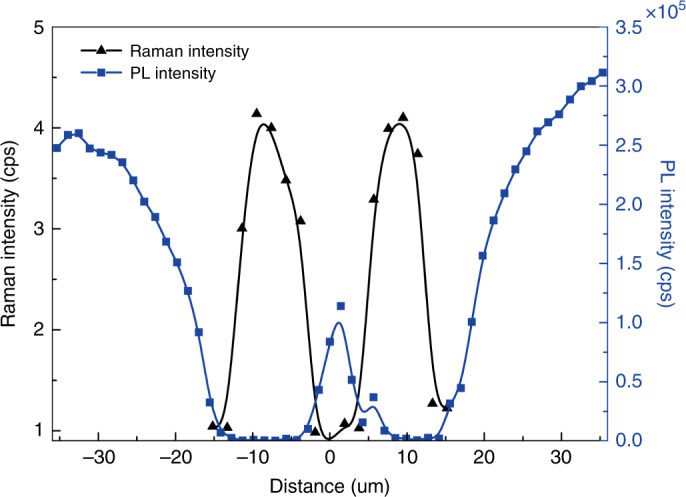
Spatial profiles of the PL and L_+_ mode intensity measured along a line, shown in Fig. 1b, passing the doublet defect in S2 (at 5.6 × 10^4^ W/cm^2^)

The data shown in Fig. 1b, e clearly show that doublet defects can be much better resolved by Raman mapping. Fig. 4 explicitly plots the intensity profiles for PL and Raman along the line passing through the two defects. Evidently, the contrast between the adjacent ends of the two defects is greatly improved in Raman, despite a DL of ~20 μm.

In summary, utilizing the nonlinear dependence of the LOPP coupled Raman mode in Raman imaging, a spatial resolution near the diffraction limit has been realized for imaging defects in a semiconductor with a carrier DL that is up to 20 times larger. We anticipate that the spatial resolution can be further improved by using sub-diffraction-limited optical excitation. This approach offers superior spatial resolution relative to the more commonly adopted PL imaging technique. Furthermore, by combining Raman imaging with PL imaging, we can obtain several elusive physical parameters, including electron and hole densities and radiative and non-radiative recombination rates in the vicinity of an individual dislocation-like defect.

## Materials and methods

Sample 1 (1-1138, S1) has a 2 µm GaAs layer sandwiched between two 50 nm GaInP layers, which are all nominally undoped with an n-type background doping level of ~5 × 10^14^ cm^−3^. Sample 2 (1-1499, S2) also has a 2 µm GaAs active layer but with 100 nm GaInP barriers. The upper half of the top barrier is doped n-type to ~5 × 10^18^ cm^−3^, and the lower half is undoped. Sample 3 (1-1366, S3) is a solar cell comprising a GaAs p-n junction with a 40 nm n-type (~10^18^ cm^−3^) emitter above a 3 µm p-type (~7 × 10^16^ cm^−3^) base. The top and bottom GaInP layers are both 50 nm thick, with n-type and p-type doping, respectively. The samples are all grown on a GaAs substrate with a GaAs buffer layer via metal-organic vapor phase epitaxy. All experiments were conducted at room temperature using a Horiba LabRAM HR800 confocal Raman microscope using a ×100 microscope objective lens (NA=0.9) and a 532 nm laser. The PL and Raman signals were acquired via a CCD detector with laser powers varying from 20 to 200 µW at the sample. The PL and Raman images were acquired in raster scan mode.
